# Selective interactions between epithelial tumour cells and bone marrow mesenchymal stem cells

**DOI:** 10.1054/bjoc.1999.1093

**Published:** 2000-03-06

**Authors:** H Hombauer, J J Minguell

**Affiliations:** Unidad de Biología Celular, INTA, Universidad de Chile, Casilla 138, Santiago, 11, Chile; Unidad de Trasplante de Médula Osea, Clínica Las Condes, Lo Fontecilla 441, Santigo, Chile

**Keywords:** breast cancer cells, bone marrow, mesenchymal stem cells, micrometastases, cell adhesion molecules, interaction

## Abstract

This work is a comparative study on the features displayed by an epithelial metastatic breast cancer cell line (MCF-7) when set in co-culture with human bone marrow mesenchymal stem cells (MSC) or a feeder layer of 3T3 fibroblasts. MSC, a subset of non-haematopoietic cells in the marrow stroma, display a potential for self-renewal, proliferation and differentiation into precursors for bone, cartilage, connective and muscular tissue. Adhesion of MCF-7 cells to monolayers of MSC or 3T3 was high (95 and 85% respectively). Once attached, MCF-7 grow well on both monolayers. Morphology of MCF-7 cells, as analysed by light and epifluorescence microscopy, revealed that MCF-7 cells grow in clusters on 3T3, but disperse on MSC. Concomitant with the lost of their aggregation status, MCF-7 on MSC express low levels of the intercellular adhesion molecules, E-cadherin and epithelial-specific antigen (ESA). These results suggest that MSC represent an appropriate cell target to investigate the cellular and molecular events occurring at the interface of epithelial-marrow stromal interactions. Together, the model here described should permit to further evaluate the significance and prognostic impact of the shift of micrometastatic cells from a cluster-aggregated into a single-cell status. © 2000 Cancer Research Campaign


*British Journal of Cancer* (2000) **82**(7), 1290–1296
© 2000 Cancer Research Campaign
DOI: 10.1054/ bjoc.1999.1093, available online at http://www.idealibrary.com on IDEAL®

### Selective interactions between epithelial tumour cells and bone marrow mesenchymal stem cells

**H Hombauer[1] and JJ Minguell[1,2]**

[1]Unidad de Biología Celular, INTA, Universidad de Chile, Casilla 138, Santiago 11, Chile; [2]Unidad de Trasplante de Médula Osea, Clínica Las Condes, Lo Fontecilla 441, Santiago, Chile

**Summary** This work is a comparative study on the features displayed by an epithelial metastatic breast cancer cell line (MCF-7) when set in co-culture with human bone marrow mesenchymal stem cells (MSC) or a feeder layer of 3T3 fibroblasts. MSC, a subset of non-haematopoietic cells in the marrow stroma, display a potential for self-renewal, proliferation and differentiation into precursors for bone, cartilage, connective and muscular tissue. Adhesion of MCF-7 cells to monolayers of MSC or 3T3 was high (95 and 85% respectively). Once attached, MCF-7 grow well on both monolayers. Morphology of MCF-7 cells, as analysed by light and epifluorescence microscopy, revealed that MCF-7 cells grow in clusters on 3T3, but disperse on MSC. Concomitant with the lost of their aggregation status, MCF-7 on MSC express low levels of the intercellular adhesion molecules, E-cadherin and epithelial-specific antigen (ESA). These results suggest that MSC represent an appropriate cell target to investigate the cellular and molecular events occurring at the interface of epithelial-marrow stromal interactions. Together, the model here described should permit to further evaluate the significance and prognostic impact of the shift of micrometastatic cells from a cluster-aggregated into a single-cell status. © 2000 Cancer Research Campaign

**Keywords**: breast cancer cells; bone marrow; mesenchymal stem cells; micrometastases; cell adhesion molecules; interaction

The fate of breast cancer patients after local curative resection depends on the capacity of the primary tumour cells to disseminate to distant organs in an early stage of cancer. The identification of tumour cell dissemination on a single-cell level, termed micrometastases, has been considered a direct approach to defining the disseminative potential of a tumour and a practical tool to identify patients at high risk for tumour recurrence (Cote et al, 1991; O'Sullivan et al, 1997).

Bone marrow represents an optimal destination site for micrometastatic breast cancer cells, however, it is not clear whether their presence in the marrow represents true residual disease, cell shedding from the primary tumour and/or metastatic potential of the primary tumour (Diel et al, 1992; Ross et al, 1993; Martin et al, 1998). Thus, the bone marrow microenvironment in addition to its role in self-renewal, commitment and maturation of the haemopoietic stem cell (Klein, 1995), seems also to be an appropriate 'niche' for homing, attachment, dormancy, modulation of growth and development of disseminated micrometastatic cells. This distinctive property of the marrow microenvironment is probably related to the competence of stromal cells to produce a combination of cytokines, extracellular matrix molecules and by their ability to establish heterotypic cell–cell contacts (Tavassoli and Minguell, 1991; Chichester et al, 1993; Klein, 1995).

While several cellular and molecular aspects of the interaction between breast epithelial cells with its surrounding mammary stroma has been well established (Sawhney et al, 1992; Hazan et al, 1997), their interaction with elements of the marrow stroma is poorly understood. Among the cellular complexity of marrow stroma exists a subset of non-haematopoietic cells referred as mesenchymal stem cells (MSC), which display a potential for self-renewal, proliferation and differentiation (Prockop, 1997; Conget and Minguell, 1999). These properties confer to the multipotential MSC the capability to serve as long-lasting precursors for bone, cartilage, connective and muscular tissue (Pereira et al, 1995; Ferrari et al, 1998). Together, MSC produce a vast array of cytokines and extracellular matrix molecules (Haynesworth et al, 1996; Prockop, 1997) and express receptors and/or counter-receptors both for cell–cell and cell–matrix interactions (Prockop, 1997; Conget and Minguell, 1999).

All these attributes make the MSC an interesting cell phenotype to investigate their potential to interact with tumour epithelial cells. The above mentioned interest is further strengthened by the observation that long-term marrow stromal cells provide an advantageous environment for the adhesion but not for the growth of mammary epithelial cells (Brooks et al, 1997). Since long-term marrow stromal cells differ from MSC in immunophenotype and multipotential capabilities (Prockop, 1997; Majumdar et al, 1998), we have initiated studies to investigate the growth pattern, morphogenetic organization and expression of cell–cell adhesion molecules in an epithelial breast cancer cell, after interacting in a co-culture system with marrow-derived mesenchymal stem cells.

*Received 6 May 1999*
*Revised 5 October 1999*
*Accepted 9 October 1999*

*Correspondence to:* JJ Minguell

### MATERIALS AND METHODS

### Cell sources

Bone marrow MSC were prepared from leftover material obtained from heparinized bone marrow samples from normal individuals

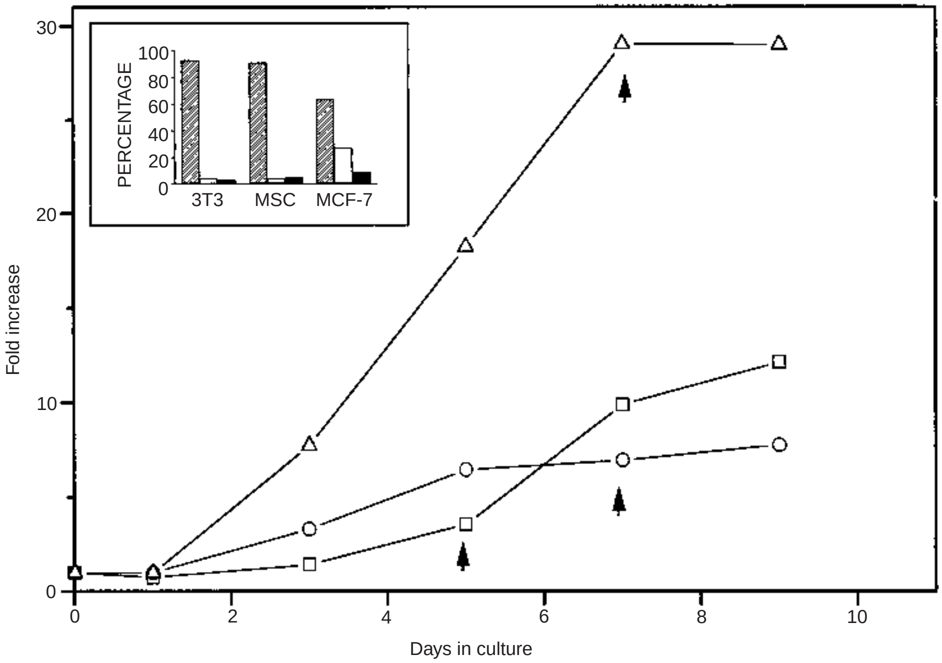

**Figure 1** Representative growth curves for MCF-7, MSC and 3T3 cells. At each culture time, the total number of 3T3 (△), MSC (○) and MCF-7 (□) cells was counted (haemocytometer chamber) and expressed as fold increase over the respective starting cell number, which was set to 1. For each cell line, at the indicated day of culture (arrow), cell cycle status was assessed by DNA content analysis. Inset shows the percentage of cells at each phase of the cell cycle (dash = G1/G0, open = S, close = G2/M)

undergoing marrow harvests for allogeneic transplantation (Satomura et al, 1998; Conget and Minguell, 1999). Briefly, marrow mononuclear cells were suspended in α-MEM (modified essential medium) containing 20% fetal calf serum (FCS; Gibco-BRL, NY, USA), seeded in T-25 flasks ($1 \times 10^6$ cells $cm^{-2}$) and cultured (37°C, 5% carbon dioxide ($CO_2$)). One week later, the evolving adherent cell layer was trypsinized (0.25% trypsin, Sigma, St Louis, MO, USA), resuspended and subcultured. Adherent cells after the third subculture, here referred to as MSC, were used for the experiments described.

The 3T3 fibroblastic cell line (ATCC, CCL 92), a competent feeder layer for studies of mesenchymal and epithelial interactions (Watt, 1994), was used as a control for co-culture studies. Cells were seeded at $5 \times 10^3$ cells $cm^{-2}$ in culture medium and incubated at 37°C in an atmosphere of 5% $CO_2$. After 1 week, the confluent monolayer was trypsinized and cells were maintained by weekly passages at 1:5 to 1:10 dilution.

MCF-7, an established human metastatic breast cancer cell line widely utilized as a test cell for studies of mammary epithelial cells and their interaction with the surrounding stroma (Ryan et al, 1993; Dong-Le Bourhis et al, 1997), was used in these studies. Cells (ATCC, HTB 22) were seeded at $10 \times 10^3$ cells $cm^{-2}$ in culture medium and incubated at 37°C in an atmosphere of 5% $CO_2$. After 1 week, adherent cells were trypsinized and weekly passaged at 1:10 to 1:20 dilution.

### Co-culture of MCF-7 cells with monolayers of MSC or 3T3 cells

Co-cultures were established by plating MCF-7 cells ($20–30 \times 10^3$ in 5 ml of culture medium) in T-25 flasks containing a confluent monolayer of MSC or 3T3 cells. At these conditions, the number of MCF-7 cells relative to total cell number in the co-cultures was 4–6%. After a 4-h incubation period, non-adherent or loosely adherent cells were eliminated by three washings with phosphate-buffered saline (PBS) containing 2% FCS. Culture medium was readded and co-cultures were incubated at 37°C for various time periods, with a change of medium every 48 h. At the completion of each incubation period, the adherent layers (of at least three T-25 flasks) were detached either by exposure to a trypsine (0.25% in 0.2 mM EDTA) or a EDTA (1 mM EDTA in PBS) solution. Detached cells in PBS–2% BSA (bovine serum albumin) and brought to a single-cell suspension by pipetting, were used for cell count (haemocytometer) and viability determination (trypan blue) and to measure the number of immunoreactive MCF-7 cells by flow cytometry.

**Table 1** Expression of E-cadherine and ESA on MCF-7 cells

| Co-culture of MCF-7 with: | E-cad (relative expression*) | ESA |
|---|---|---|
| 3T3 | 1.00 | 1.00 |
| MSC | 0.42 | 0.36 |

Co-cultures of MCF-7 with 3T3 or MSC cells (day 4) were analysed for E-cadherine (E-cad) and ESA expression, as indicated under Materials and Methods. *For each condition, the mean fluorescence intensity of each antigen was measured and relative values calculated with respect to MFI for E-cad and ESA expression in co-cultures with 3T3, which were set to 1. Data shown are representative of two experiments in duplicate, with s.d. values always less than 10%.

© 2000 Cancer Research Campaign 

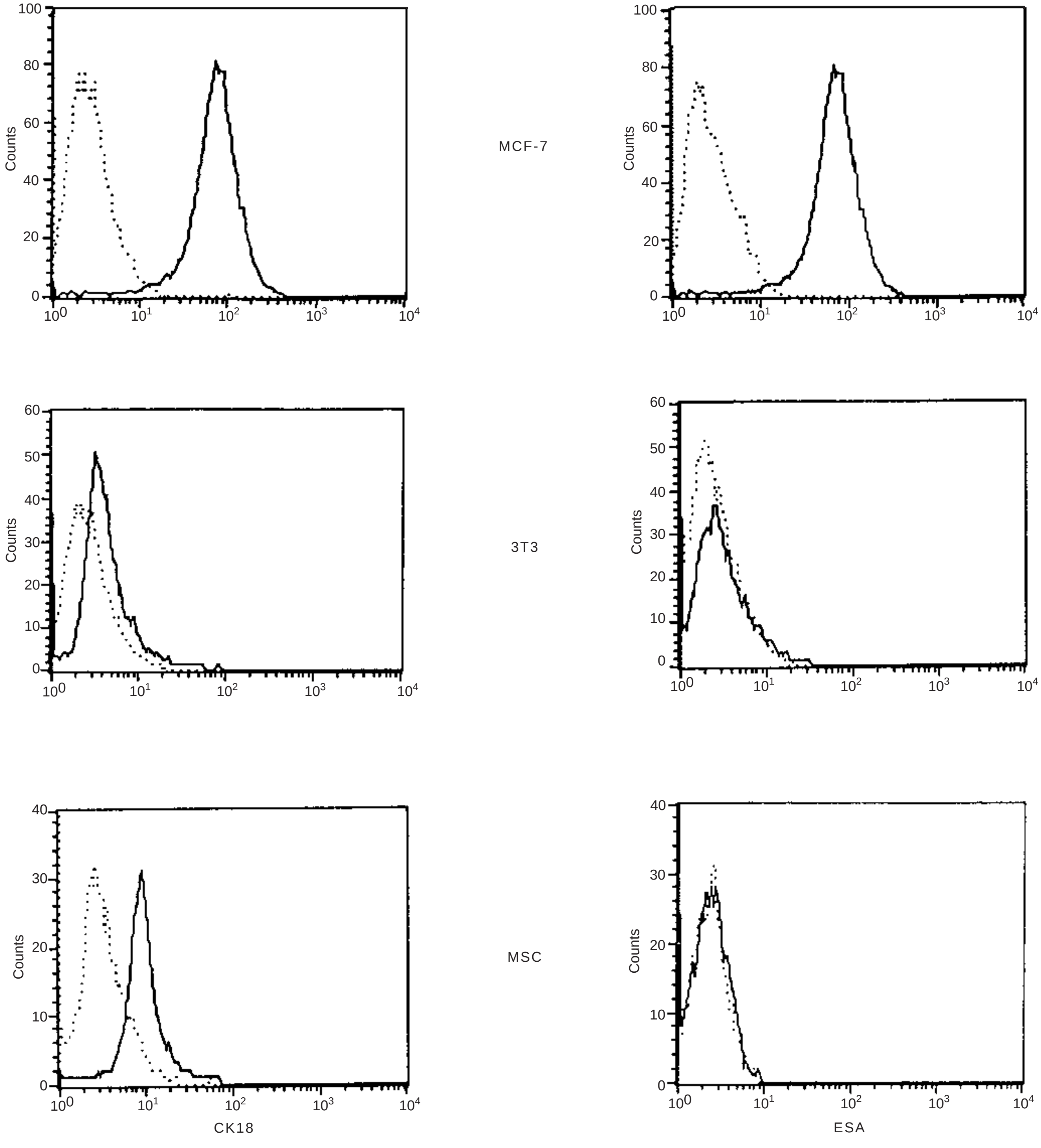

**Figure 2** Expression of antigens CK18 and ESA in MCF-7, 3T3 and MSC cells. Relative number of cells is presented versus fluorescence intensity. The fluorescence profile (log scale) of unstained cells (dotted line) is compared with the same cell suspension after labelling with the indicated antibody (solid line). These data are representative of four experiments

### Antibody staining

To detect the intracellular antigen, cytokeratin peptide 18 (CK18), cells were permeabilized (70% ethanol, 10 min, 4°C), and incubated (30 min at 4°C) with fluorescein isothiocyanate (FITC)-conjugated anti-cytokeratin peptide 18 (CK18) monoclonal antibody (Sigma). As isotype control, FITC-conjugated mouse $IgG_1$ antibody (Becton Dickinson, San José, CA, USA) was used. To

 *© 2000 Cancer Research Campaign*

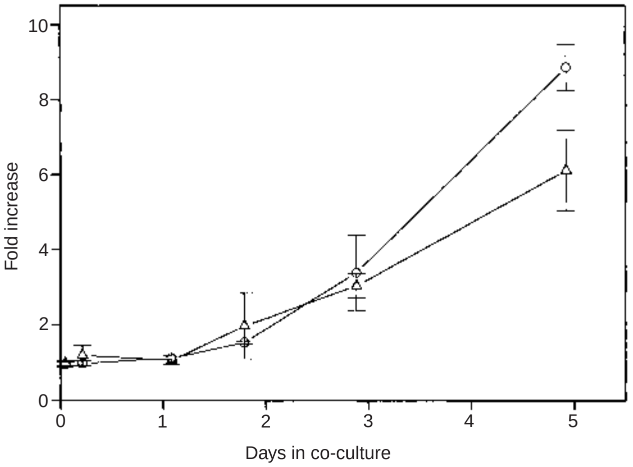

**Figure 3** Proliferation of MCF-7 cells in co-culture with monolayers of 3T3 and MSC cells. MCF-7 cells were seeded on a confluent monolayer of MSC (○) or 3T3 (△) cells. At the indicated culture time, cells were stained for CK18 and immunoreactive cells were enumerated by flow cytometry. The number of $CK18^+$ cells in the co-culture was calculated and expressed as fold increase over the respective number of seeded MCF-7 cells, which was set to 1. Each data point represents the mean ± s.e.m. of at least three experiments detect the surface-associated epithelial-specific antigen (ESA), cells were incubated with anti-human ESA monoclonal antibody (Sigma), followed by incubation with FITC-conjugated antimouse IgG (wm) antibody (Sigma). For CK18 and ESA studies, the concentration of the primary antibody for the cell number used was previously established by titration. For E-cadherin (E-Cad) staining, cells were labelled with an anti-rat uvomorulin (L-CAM) monoclonal antibody (Sigma) and further incubated with a FITC-conjugated anti-rat IgG antibody (Sigma). Flow cytometric analysis was performed using a FACScan flow cytometer (Beckton Dickinson), using the CELLQUEST software. Usually, 10 000 events were obtained for analysis, although for co-culture analysis, 40 000 events were obtained. For comparative purposes, the mean fluorescence intensity (MFI) of each antigen is expressed as the ratio of MFI for first antibody/MFI for control antibody.

### Cell cycle analysis

For these studies, cells were permeabilized, labelled with 10 μg $ml^{-1}$ propidium iodine (Pl; Sigma), and treated with 0.1 mg $ml^{-1}$ RNAase (Sigma). DNA content was analysed in a FACScan flow cytometer, using the ModFIT software. Usually, 10 000 events were obtained for analysis.

### Microscopic evaluation of MCF-7 cells in co-culture

The organization status of MCF-7 cells in co-culture was evaluated either by phase-contrast or by epifluorescence microscopy after labelling with anti-CK18, anti-ESA or anti-E-Cad antibodies, as indicated above. As seen under light or epifluorescence microscopy, a cluster was defined as consisting of more than six immunoreactive cells, in direct cell–cell contact without any intercellular space.

### RESULTS

### Growth and cell cycle status of MCF, MSC and 3T3 cells

To define proper conditions for the co-culture experiments, each cell type was set in culture and assessed for growth and cell cycle status (Figure 1). While 3T3 and MSC cells grow and attained a typical confluence growth-arrested phase (at around day 7), MCF-7 cells reach a rapid semi-confluence condition (day 5), beyond which proliferation still occurs, but at a lower rate. The above was further documented by DNA content analysis, which show that by day 7 of culture more than 90% of MSC and 3T3 cells were in the G0/G1 phase of the cell cycle (Figure 1, inset). For MCF-7 (day 5), the fraction of cells at G0/G1 was 60%, being the rest of cells at S or G2/M.

### Detection of immunoreactive MCF-7 cells by flow cytometry

To validate a method to distinguish and enumerate immunoreactive MCF-7 cells, particularly for the co-culture experiments, titration studies were performed to establish a proper concentration of antibody (anti-CK18 or anti-ESA) that assures a high fluorescence signal for MCF-7 and a low or meaningless signal for MSC and 3T3 cells. As seen in Figure 2, at the antibody concentration selected (1/400 and 1/800 dilution for CK18 and ESA respectively), MCF-7 cells express both antigens with high fluorescence intensities (MFI ≥ 40), whereas the expression of both antigens in MSC and 3T3 cells was negligible (MFI ≤ 2). To further validate the immunofluorescence method to enumerate MCF-7 cells, MSC or 3T3 cells were mixed at different proportions (2–15%) with MCF-7. After labelling with anti-CK18 antibody and enumeration of immunoreactive cells by flow cytometry, the average recovery of MCF-7 cells was 97 ± 6% ($n$ = 7).

### Proliferation of MCF-7 cells in co-culture with monolayers of MSC or 3T3 cells

Co-cultures were established by seeding MCF-7 cells (see Figure 1, arrow) on top of monolayers of 3T3 or MSC cells (see Figure 1, arrows). Results in Figure 3 show the time-dependent accumulation of immunoreactive ($CK18^+$) MCF-7 cells on each monolayer. It can be seen that the growth of MCF-7, after 5 days in co-culture, is not significantly different beween both monolayers. Similar results were obtained when MCF-7 cells in the co-culture were labelled with anti-ESA antibody to track their proliferation (data not shown). Longer co-culture periods (> 6 days) were not analysed, due to detachment of the MSC or 3T3 monolayer from the culture vessels.

### Aggregation status of MCF-7 cells in co-culture with MSC or 3T3 cells

To investigate the morphological features of MCF-7 cells in co-culture, cells were observed in situ under phase-contrast or by epifluorescence microscopy after labelling with anti-CK18, anti-ESA or anti-E-Cad antibodies. As visualized under phase-contrast microscopy, MCF-7 cells either in clusters or as single cells appear to be attached to all over the fibroblastoid-like cells forming the 3T3 and MSC monolayers respectively (Figure 4, A, B). In co-culture with 3T3 cells, immunoreactive MCF-7 cells were

© 2000 Cancer Research Campaign

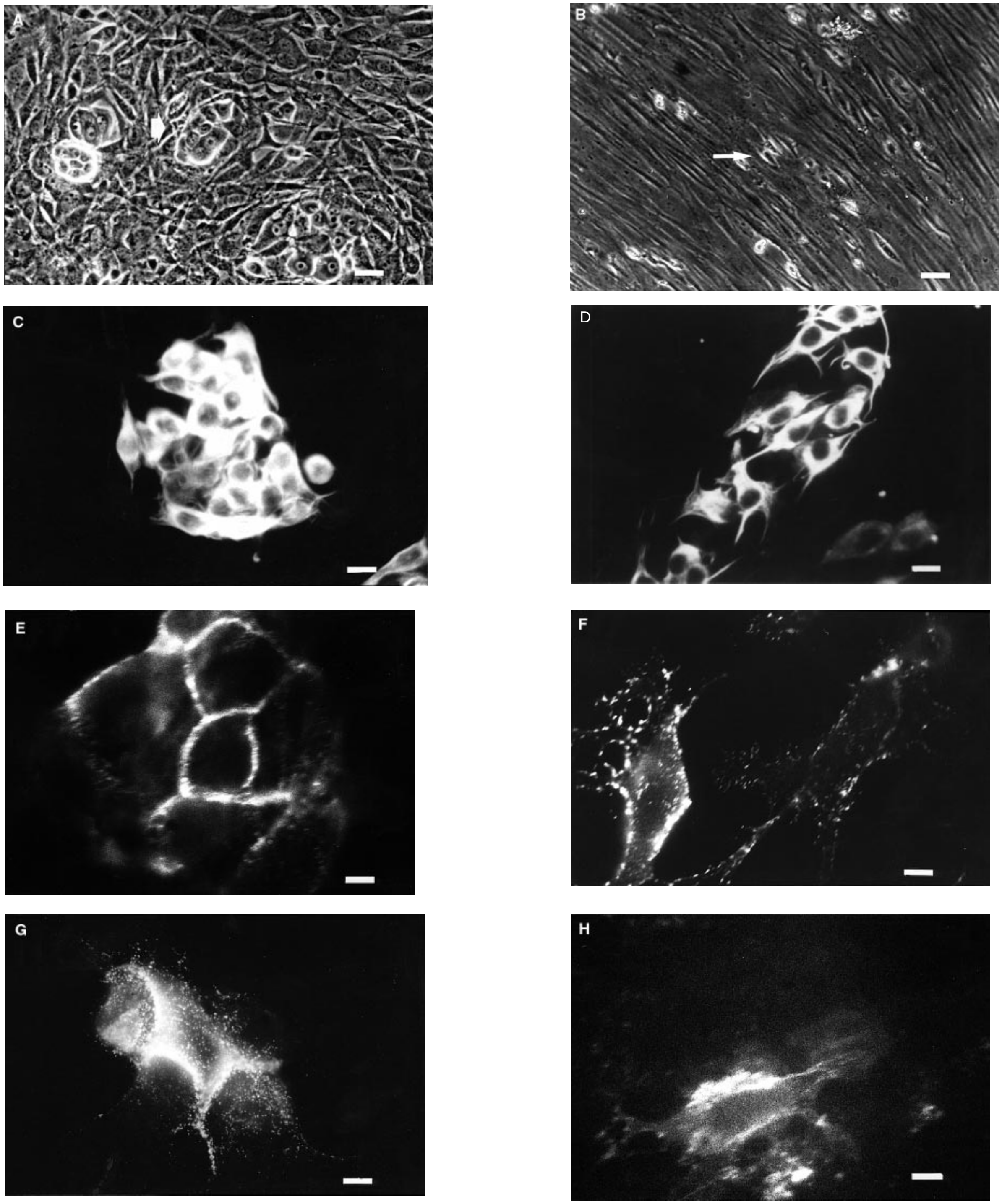

**Figure 4** Photomicrographs showing clustered or single MCF-7 cells in co-culture with 3T3 and MSC cells. Photomicrographs were taken on co-cultures (4 days) of MCF-7 cells with 3T3 (left) or MSC (right) cells. Panels **A** and **B** show unstained cells, as visualized under phase-contrast microscopy. Arrows indicate clustered (➧) or single (→) cells. Other panels show immunoreactive MCF-7 cells after staining with antibodies against: CK18 (**C** and **D**), ESA (**E** and **F**) and E-Cadherin (**G** and **H**). Notice that for co-cultures with MSC and after labelling with anti-E-Cad (**H**), since the fluorescence signal is weak, the film was over-exposed to get the microphotograph. Scale bars: 70 µm (**A, B**); 35 µm (**C, D, E, F**); 14 µm (**G, H**)

 *© 2000 Cancer Research Campaign*

visualized as growing in clusters of > 6 round cells in direct cell–cell contact and without any visible intercellular space (Figure 4, C, E and G). Small clusters (2–5 cells) and few single immunoreactive cells were only seen at the initial stages of the co-culture. Conversely, in co-culture with MSC, immunoreactive MCF-7 cells have lost their aggregation status and are visualized as single cells, even after prolonged periods of co-culture (Figure 4, D, F and H).

### Expression of intercellular adhesion molecules in MCF-7 cells in co-culture

The expression of E-cadherine and ESA on MCF-7 cells in co-culture was assessed to investigate whether changes in the expression of the adhesion molecules, may explain why MCF-7 grow in cluster on 3T3 but disperse on MSC. As shown in Table 1, the relative expression of both intercellular adhesion molecules was 60% lower in MCF-7 cells in co-cultures with MSC than with 3T3 cells.

### DISCUSSION

The degree to which micrometastases within the bone marrow of patients with breast cancer represents true residual disease, cell shedding and/or metastatic potential is unclear (Funke et al, 1996; O'Sullivan et al, 1997). In addition, the correlation between the presence of epithelial tumour cells in mesenchymal cell samples (blood or bone marrow), with prognosis or other clinical and pathological features, has also been controversial. While some authors have reported that finding one tumour cell among $10^6$ bone marrow cells is an independent prognostic factor for a higher incidence of recurrent metastatic disease (Cote et al, 1991; Martin et al, 1998), others have found no such correlation (Singletary et al, 1991; Molino et al, 1997). Whether the discrepancy reflects diversity in patient disease status, selection of methodological procedures to detect and enumerate cancer cells or in the origin of cancer cells in the marrow (micrometastases vs trapped circulating tumour cells) is a matter not yet established. However, an additional interpretation may be found in the notion that the outcome of the micrometastatic cell depends on the nature and status of the interacting stromal counterpart (Adam et al, 1994; Hazan et al, 1997).

In this vein, we have investigated whether marrow mesenchymal cells provide the tumour cell with an advantageous environment for adhesion, proliferation and morphogenetic organization. MSC, based on their self-renewal, proliferative and differentiation potential (Prockop, 1997; Conget and Minguell, 1999), appear as a suitable cell-target for epithelial tumour cells. With the model system here used, we found that after interacting with MSC, the breast cancer cell line MCF-7 displayed the following features:

1. MCF-7 cells once attached to the monolayer of MSC, start to proliferate at a rate that permits an eightfold increase in cell number in about 5 days. However, proliferation of MCF-7 on MSC was not better than on a feeder layer of 3T3 cells. These data concur with previous findings showing that regardless the tissue source, stromal cells stimulated the growth of MCF-7 (van Roozendaal et al, 1992) or breast cancer-derived epithelial cells (Brooks et al, 1997).
2. The microscopic evaluation of MCF-7 cells in co-culture with MSC, revealed that more than 80% of MCF-7 were present as single cells lying close together without any evidence of direct cell–cell contacts. This lack of organization contrasts with the aggregation status exhibited by MCF-7 cells in co-culture with 3T3 or cultured on an inert plastic surface (not shown). Under these conditions, MCF-7 cells grow in well organized clusters exhibiting cell–cell contacts and few intercellular spaces. Our results are different to those reported by Brooks et al (1997), who showed that primary cultures of epithelial cells once attached to a monolayer of long-term marrow stroma, proliferates and give rise to clusters (colonies) of epithelial cells. Despite that MSC and long-term marrow stromal cells are both considered as 'stromal cells', the evolving phenotypes, the differentiation potential and secretion products (cytokines and matrix molecules), are quite different (Chichester et al, 1993; Prockop, 1997; Majumdar et al, 1998; Conget and Minguell, 1999). On the other hand, our results related to the transition of clustered to single MCF-7 cells, resemble the appearance of micrometastatic cells in the marrow of breast cancer patients. Thus, it has been reported that when the load of immunoreactive cancer ($CK18^+$) cells is low, single cells are detectable in the marrow, whereas as their numbers increase, cell clusters predominate (Funke et al, 1996; Müller et al, 1996).
3. The expression of two main epithelial intercellular adhesion molecules, E-cad and ESA, seems to be down-regulated in MCF-7 cells in co-culture with MSC, as compared to 3T3. We speculate that the extent of E-cad and ESA down-regulation is sufficient to avoid homotypic cell adhesion; hence single and not clustered MCF-7 cells develop on MSC. Our observation of single breast cancer epithelial cells with low expression of E-cad is not without precedent. In micrometastatic cells derived from breast cancer patients, such cells are present (Funke et al, 1996) and are probably derived from a primary tumour with an elevated invasive potential (Takeichi, 1991; Funke et al, 1996).

Taken together, the results here reported indicate that the interaction of MCF-7 cells with human bone marrow mesenchymal stem cells, confers the tumour cell distinctive features that may define their outcome in the marrow. The co-culture system used here will allow further investigations towards a better understanding of the cellular and molecular events occurring at the interface of epithelial–mesenchymal interactions. In addition it should permit us to evaluate the significance and prognostic impact of the shift of micrometastatic cells from a cluster-aggregated to a single-cell status (Funke et al, 1996; Muller et al, 1996, Frixen et al, 1998).

### ACKNOWLEDGEMENTS

We thank Drs Valeska Simon and JF Santibañez for valuable discussions. This work was supported by grants from FONDECYT (Chile) # 89700–28 and from the International Centre for Genetic Engineering and Biotechnology (Italy) # CRP/CHI97–01 (a1).

### REFERENCES

Adam L, Crepin M, Lelong JC, Spanakis E and Israel L (1994) Selective interactions between mammary epithelial cells and fibroblasts in co-culture. *Int J Cancer* **59**: 262–268

*© 2000 Cancer Research Campaign* 

Brooks B, Bundred NJ, Howell A, Lang SH and Testa NG (1997) Investigation of mammary epithelial cell–bone marrow stroma interactions using primary human cell culture as a model of metastasis. *Int J Cancer* **73**: 690–696

Chichester CO, Fernández M and Minguell JJ (1993) Extracellular matrix gene expression by human bone marrow stroma and by marrow fibroblasts. *Cell Adh Comm* **1**: 93–99

Conget PA, Minguell JJ (1999) Phenotypical and functional properties of human bone marrow mesenchymal progenitor cells. *J Cell Physiol* **181**: 67–73

Cote RJ, Rosen PP, Lesser ML, Old LJ, Osborne MP (1991) Prediction of early relapse in patients with operable breast cancer by detection of occult bone marrow micrometastases. *J Clin Oncol* **9**: 1749–1756

Diel IJ, Kaufmann M, Goerner R, Costa SD, Kaul S, Bastert G (1992) Detection of tumor cells in bone marrow of patients with primary breast cancer: a prognostic factor for distant metastasis. *J Clin Oncol* **10**: 1534–1539

Dong-Le Bourhis X, Berthois Y, Millot G, Degeorges A, Sylvi M, Martin PM, Calvo F (1997) Effect of stromal and epithelial cells derived from normal and tumours breast tissue on the proliferation of human breast cancer cell lines in co-culture. *Int J Cancer* **71**: 42–48

Ferrari G, Cusella-de Angelis G, Coletta M, Paolucci E, Stomaiuolo A, Cossu G, Mavilio F (1998) Muscle regeneration by bone marrow-derived myogenic progenitors. *Science* **279**: 1528–1530

Frixen UH, Behrens J, Sachs M, Eberle G, Voss B, Warda A, Lochner D, Birchmeier W (1998) E-cadherin-mediated cell–cell adhesion prevents invasiveness of human carcinoma cells. *Exp Cell Res* **240**: 420–432

Funke I, Fries S, Rolle M, Heiss M, Untch M, Bohmert H, Schildberg FW, Jauch KW (1996) Comparative analyses of bone marrow micrometastases in breast and gastric cancer. *Int J Cancer* **65**: 755–761

Haynesworth SE, Baber MA, Caplan AI (1996) Cytokine expression by human marrow-derived mesenchymal progenitor cells in vitro: effects of dexamethasone and IL-1a. *J Cell Physiol* **166**: 585–592

Hazan RB, Kang L, Whooley BP, Borgen PI (1997) N-cadherin promotes adhesion between invasive breast cancer cells and the stroma. *Cell Adhes Commun* **4**: 399–411

Klein G (1995) The extracellular matrix of the hematopoietic microenvironment. *Experientia* **51**: 914–926

Majumdar MK, Thiede MA, Mosca JD, Moorman M, Gerson SL (1998) Phenotypic and functional comparison of cultures of marrow-derived mesenchymal stem cells (MSCs) and stromal cells. *J Cell Physiol* **176**: 57–66

Martin VM, Siiewert C, Scharl A, Harms T, Heinze R, Öhl S, Radbruch A, Miltenyi S, Schmitz J (1998) Immunomagnetic enrichment of disseminated epithelial tumor cells from peripheral blood by MACS. *Exp Hematol* **26**: 252–264

Molino A, Pelosi G, Turazza M, Sperotto L, Bonetti A, Nortilli R, Fattovich G, Alaimo C, Piubello Q, Pavenel F, Micciolo R, Cetto GL (1997) Bone marrow micrometastases in 109 breast cancer patients: correlations with clinical and pathological features and prognosis. *Breast Cancer Res Treat* **42**: 23–30

Müller P, Weckermann D, Riethmüller G, Schlimok G (1996) Detection of genetic alterations in micrometastatic cells in bone marrow of cancer patients by fluorescence in situ hybridization. *Cancer Genet Cytogenet* **88**: 8–16

O’Sullivan GC, Collins JK, Kelly J, Morgan J, Madden M, Shanahan F (1997) Micrometastases: marker of metastatic potential or evidence of residual disease. *Gut* **40**: 512–515

Pereira RF, Halford KW, O’Hara MD, Leeper DB, Sokolov BP, Pollard MD, Bagasra O, Prockop DJ (1995) Cultured adherent cells from marrow can serve as long-lasting precursor cells for bone, cartilage, and lung in irradiated mice. *Proc Natl Acad Sci USA* **92**: 4857–4861

Prockop DJ (1997) Marrow stromal cells as stem cells for non-hematopoietic tissues. *Science* **276**: 71–74

Ross AA, Cooper BW, Lazarus HM, Mackay W, Moss TJ, Ciobanu N, Tallman MS, Kennedy MJ, Davidson NE, Sweet D, Winter C, Akard L, Jansen J, Copelan E, Meagher RC, Herzig RH, Klumpp TR, Kahn DG, Warner NE (1993) Detection and viability of tumor cells in peripheral blood stem cell collections from breast cancer patients using immunocytochemical and clonogenic assay techniques. *Blood* **82**: 2605–2610

Ryan MC, Orr DJA, Horgan K (1993) Fibroblast stimulation of breast cancer cell growth in a serum-free system. *Br J Cancer* **67**: 1268–1273

Satomura K, Derubeis AR, Fedarko NS, Ibaraki-O’Connor K, Kuznetsov SA, Rowe DW, Young MF, Gehron Robey P (1998) Receptor tyrosine kinase expression in human bone marrow stromal cells. *J Cell Physiol* **177**: 426–438

Sawhney N, Garrahan N, Douglas-Jones AG, Williams ED (1992) Epithelial-stromal interactions in tumors. A morphologic study of fibroepithelial tumors of the breast. *Cancer* **70**: 2115–2120

Singletary SE, Larry L, Tucker SL, Spitzer G (1991) Detection of micrometastatic tumor cells in bone marrow of breast carcinoma patients. *J Surg Oncol* **47**: 32–36

Takeichi M (1991) Cadherin cell adhesion receptors as a morphogenetic regulator. *Science* **251**: 1451–1455

Tavassoli M, Minguell JJ (1991) Homing of hemopoietic progenitor cells to the marrow. *Proc Soc Exp Biol Med* **196**: 367–373

van Roozendaal CP, Van Oojen B, Klijn JMG, Classen C, Eggermont AMM, Henzen-Logmans SC, Foekens JA (1992) Stromal influences on breast cancer cell growth. *Br J Cancer* **65**: 77–81

Watt FM (1994) Cultivation of human epidermal keratinocytes with a 3T3 feeder layer. In: *Cell Biology, A Laboratory Handbook*, Celis JE (ed), pp. 83–89. Academic Press: San Diego

 *© 2000 Cancer Research Campaign*
